# Understanding the relationships among dispositional mindfulness, PsyCap, creative motivation, and creative performance: a sequential mediation model

**DOI:** 10.3389/fpsyg.2025.1605358

**Published:** 2025-07-17

**Authors:** Wu-jing He, Kai Zhang

**Affiliations:** Department of Special Education and Counselling, The Education University of Hong Kong, Tai Po, Hong Kong SAR, China

**Keywords:** creativity, dispositional mindfulness, positive psychology, psychological capital, motivation, mediation analysis

## Abstract

Drawing on insights from positive psychology theory and conservation of resources (COR) theory, the present study explored how dispositional mindfulness affects creativity through the sequential mediating effect of two positive psychological constructs—psychological capital (PsyCap) and creativity motivation. A total of 658 undergraduates in Hong Kong (51.3% female; *M*_age_ = 20.9 years) completed the study. Dispositional mindfulness, PsyCap, and creativity motivation were measured via the Chinese versions of the Mindful Attention Awareness Scale, the revised Compound PsyCap Scale, and the Creativity Motivation Scale, respectively. Regarding creativity, a multidimensional assessment approach was used to evaluate creative performance across three dimensions—idea production, creative combination, and restructuring problem solving—by using three creativity tests: (1) a divergent thinking test, (2) a gestalt combination test, and (3) a creative problem-solving test. Mediation analyses revealed that PsyCap and creativity motivation partially but significantly mediated the effect of dispositional mindfulness on all three dimensions of creativity as sequential mediators, while the residual direct effect suggested the possibility of additional unexamined pathways. These findings shed light on the psychological mechanism of positive human functioning in relation to positive personal attributes, psychological resources, and creative functioning.

## 1 Introduction

The link between dispositional mindfulness and creativity has attracted increasing attention from researchers (Hughes et al., 2023). Dispositional mindfulness is defined as the ability to focus on nonjudgmental awareness in the moment (Schutte and Malouff, 2023), and creativity is understood as the ability to produce ideas, solutions, and behaviors that are considered novel and appropriate (Sternberg et al., 2024). While many studies have supported a direct link between these two constructs (He, 2023a; Henriksen et al., 2020), recent studies have focused on uncovering the mechanisms underpinning this relationship (He, 2024). Extending this line of research, the present study proposes and investigates a chain mediation mechanism by drawing on insights from positive psychology theory and conservation of resources (COR) theory, which suggest that dispositional mindfulness impacts creativity via the sequential mediating effect of two positive personal attributes—psychological capital (PsyCap) (mediator 1) and creativity motivation (mediator 2).

### 1.1 PsyCap as a mediator

According to positive psychology theory (Seligman and Csikszentmihalyi, 2000), dispositional mindfulness is an important positive personal characteristic that promotes effective human functioning, including creativity (Sanchez et al., 2023; Tsai et al., 2024). Empirical evidence has demonstrated positive correlations between dispositional mindfulness and various dimensions of creative performance, such as fluency and flexibility in divergent production, innovative and boundary-breaking thinking in creative combination, and restructuring resolution in solving problems that require creative insights (He, 2023a, 2024; Henriksen et al., 2020). Empirical evidence has also shown that dispositional mindfulness contributes to important thinking abilities that may facilitate creativity, such as open-minded thinking (openness to accepting new ideas; Deng et al., 2012), nonhabitual thinking (a tendency to generate atypical and novel initiatives; Henriksen et al., 2020), and inquisitive thinking (a desire to explore and uncover new knowledge; Grzybowski and Brinthaupt, 2022).

To clarify the underlying mechanism of the mindfulness–creativity relationship, positive psychology theory postulates that positive trait characteristics (e.g., dispositional mindfulness) may promote promising functional outcomes (e.g., creativity) by facilitating a positive psychological state characterized by PsyCap, which is defined as positive psychological strengths and resources (He, 2024; Luthans et al., 2007). PsyCap is understood as a positive construct that consists of four subdimensions, which are described as HERO characteristics (Lorenz et al., 2022), including (1) hope (i.e., a goal-oriented determination for success); (2) self-efficacy (i.e., self-confidence in personal success); (3) resilience (i.e., the ability to overcome and endure difficult situations); and (4) optimism (i.e., a positive outlook and attributions about success). In positive psychology, PsyCap is facilitated by positive personal attributes such as mindfulness and provides positive psychological strengths, capacities, and resources to promote effective human functioning, such as creativity (Corbu et al., 2022; Luthans et al., 2024). Briefly, this theoretical perspective suggests a mindfulness–PsyCap–creativity association.

Consistent with this theoretical perspective, research findings indicate that dispositional mindfulness is positively associated with all four subdimensions of PsyCap (Gordani and Sadeghzadeh, 2023). Research findings have also revealed positive connections between all four subdimensions of PsyCap and improved creativity. Moreover, research findings have shown that as a higher-order construct, PsyCap is a stronger predictor of creativity than any of its individual subcomponents are (Li et al., 2023; Luthans et al., 2024). Recently, He (2024) reported direct empirical evidence that supports the mediating effect of PsyCap on the mindfulness–creativity link via three creativity tasks that revealed distinct creative abilities, namely, divergent production, creative combination, and restructuring problem solving.

### 1.2 Motivation as a subsequent mediator

To further explain the mechanism by which PsyCap can lead to enhanced creative outcomes, positive psychology theory postulates a motivational pathway through which PsyCap promotes positive functioning outcomes by activating motivational resources (Lorenz et al., 2022; Luthans et al., 2007). Luthans (2015) articulated that PsyCap contributes to positive functioning by enabling positive evaluation of circumstances and the probability of success, which encourages further motivated effort and perseverance for task performance and goal achievement. In other words, PsyCap is facilitated by positive traits such as dispositional mindfulness and increases task-related motivation, facilitating improved functional outcomes by providing positive psychological strengths and resources (Li et al., 2023). Hence, PsyCap is highlighted as a personal resource that can be used to predict functional outcomes via its role in initiating and sustaining task motivation. Indeed, this theoretical notion is consistent with the prediction of conservation of resources (COR) theory (Hobfoll et al., 2018), a motivational theory that highlights that motivational factors are supported by personal resources, producing a pivotal effect on human functioning (Blasco-Giner et al., 2025). COR theory suggests that personal resources have a motivational function to support the attainment of goals, personal growth and self-development. People with more personal resources (i.e., stronger PsyCap) are motivated to work harder to cope with difficulties, meet challenges, and achieve desired goals to gain more resources (Hobfoll et al., 2018). In contrast, people with fewer resources (i.e., weaker PsyCap) seek to protect the resources they already possess and therefore tend to exhibit negative working attitudes and behaviors with lower motivation and engagement (Wu and Lee, 2020). Briefly, these perspectives propose a mechanism in which PsyCap and motivation function as series mediators in the mindfulness–PsyCap–motivation–creativity association.

In line with these theoretical arguments, research findings have shown that PsyCap bolsters motivation, engagement and achievement among students in the Philippines (Datu et al., 2018). Research findings also revealed that the higher the PsyCap level is, the greater the intrinsic motivation expressed by employees (Fidelis et al., 2021). More recently, preliminary empirical evidence has revealed the mediating mechanism of motivation underlying the relationships among mindfulness, PsyCap, and creative outcomes. For instance, Tran et al. (2021) reported that intrinsic motivation significantly mediated the relationship between PsyCap and innovative performance in a sample of university lecturers in southern Vietnam. Blasco-Giner et al. (2025) reported that PsyCap positively improved innovative work behavior through the mediating effect of task motivation in a sample of employees in organizational settings. Focusing on the relationships among four constructs—mindfulness, PsyCap, creative process engagement, and creativity—Li et al. (2023) reported that PsyCap partially yet significantly mediated the impact of employees' trait mindfulness on their creativity by fostering creative engagement behaviors. In short, these studies provide a theoretical and empirical basis for a mindfulness–PsyCap–motivation–creativity link.

### 1.3 Present study

**Figure 1:**
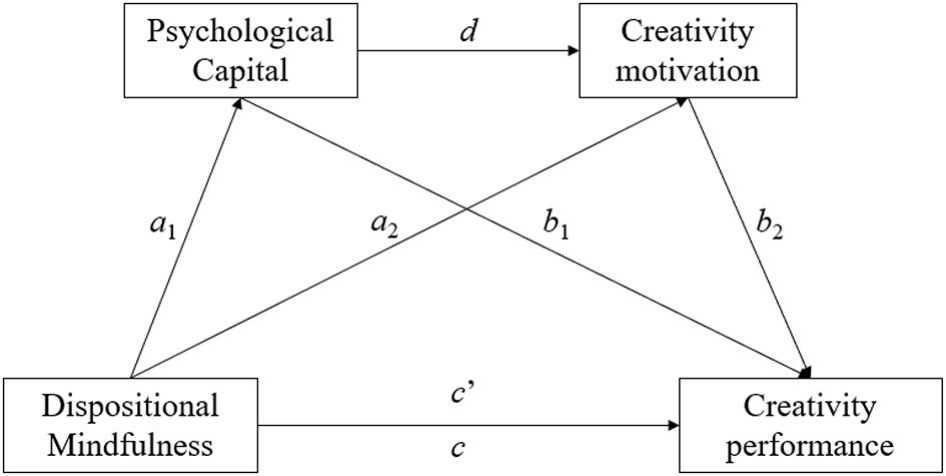
Hypothesized sequential mediation model.

The current study aims to contribute to the mindfulness–creativity field in two important ways. First, numerous studies have established direct associations between (1) dispositional mindfulness and creativity, (2) dispositional mindfulness and PsyCap, (3) PsyCap and motivation, (4) PsyCap and creativity, and (5) motivation and creativity. In addition, other studies have supported the indirect effects among these variables, where (1) PsyCap mediates the link between mindfulness and creativity (He, 2024), (2) motivation mediates the link between PsyCap and creativity (Blasco-Giner et al., 2025; Tran et al., 2021), and (3) PsyCap and task engagement work in a sequential manner to mediate the link between mindfulness and creativity (Li et al., 2023). Drawing upon these distinct lines of research, a sequential mediating effect of PsyCap (mediator 1) and motivation (mediator 2) in linking dispositional mindfulness (as the predictor variable) and creativity (as the outcome variable) is anticipated. See Figure 1 for the hypothesized mediation model. We aimed to examine these hypothesized mediation relationships in the present study; by integrating the concerted effect of these intricate relationships within one comprehensive model, we aimed to explore the fundamental process driving the mindfulness–creativity connection by taking integrative insights from positive psychology theory and COR theory.

Second, building on the multidimensional assessment approach to creativity (Sternberg et al., 2024), we followed the methodology used by He (2023a, 2024) and applied Antonietti and Iannello's (2008) taxonomy to assess three dimensions of creative performance (i.e., idea generation, combinatory ability, and restructuring ability) via corresponding creativity tests (Haase et al., 2018). First, idea production was measured via the Divergent Thinking Test [Wallach-Kogan Creativity Test (WKCT); Wallach and Kogan, 1965], a well-established tool that assesses the aspect of creativity associated with divergent production (He and Wong, 2021). Second, creative combination was evaluated with the Test for Creative Thinking–Drawing Production (TCT–DP; Urban and Jellen, 1996), which is grounded in gestalt psychology and reflects the capacity to integrate disassociated and unconnected fragments into a coherent and holistic product in an innovative and appropriate way (He and Wong, 2022). Finally, restructuring problem solving was measured through the creative problem-solving test (CPST; He and Wong, 2021), which captures the ability to generate a novel and suitable solution by reinterpreting and reframing a given problem (Weisberg, 2015). Building on the frameworks of positive psychology theory and COR theory, as well as the multidimensional assessment approach to creativity, we hypothesize that the sequential mediating model depicted in Figure 1 is supported in three dimensions of creative performance: (1) idea production, as measured by the WKCT (Hypothesis 1); (2) creative combination, as measured by the TCT–DP (Hypothesis 2); and (3) restructuring problem solving, as measured by the CPST (Hypothesis 3).

## 2 Method

### 2.1 Participants and procedures

A cross-sectional study was carried out at three Hong Kong universities via a convenience sampling method. The sample consisted of 658 undergraduates (51.3% females) who majored in education, sciences, health sciences, social sciences, linguistics, or arts. The mean age and average duration of education of the sample were 20.9 years (*SD* = 1.58; range = 18–23 years) and 14.7 years (*SD* = 1.93; range = 13–16 years), respectively. All the participants were of Chinese ethnicity. Data collection was carried out by trained researchers who were blinded to the research objectives and hypotheses. Prior to participation, all individuals were informed about the study's voluntary nature, safety measures, and confidentiality principles. During data collection, the assessments for all the study variables (i.e., dispositional mindfulness, PsyCap, motivation, creativity) were administered to the participants in a group setting of ~20–25 participants following standard instructions. Moreover, demographic data such as age, gender, and level of education were also collected. Typically, ~50–60 min were needed to complete the entire assessment procedure.

### 2.2 Instruments

#### 2.2.1 Dispositional mindfulness

Dispositional mindfulness was measured via the 15-item Chinese version of the Mindful Attention Awareness Scale (MAAS; Brown and Ryan, 2003; Deng et al., 2012), a widely recognized and well-validated tool for assessing trait mindfulness that has strong psychometric properties (Molina-Rodríguez et al., 2023). The participants rated their general awareness tendencies in daily life on a 6-point scale (1 = almost never to 6 = almost always). An example item is “I find myself preoccupied with the future or the past” (reverse coded). Higher total scores reflect greater trait mindfulness. The reliability and validity of the MAAS have been supported in both Hong Kong and mainland Chinese student populations (He, 2023a, 2024; Li et al., 2023). In the current sample, the scale exhibited good internal consistency (α = 0.89). The results of confirmatory factor analysis (CFA) revealed good fit indices for a one-factor model (*CFI* = 0.938, *TLI* = 0.921, *RMSEA* = 0.047, *SRMR* = 0.066), supporting the construct validity of the scale.

#### 2.2.2 PsyCap

PsyCap was measured via the adapted Chinese version of the revised Compound Psychological Capital Scale (CPC-12R; Lorenz et al., 2022; He, 2024). This instrument comprises 12 items, where sample items include “I can think of many ways to reach my current goals” (hope), “I am confident that I could deal efficiently with unexpected events” (self-efficacy), “After serious life difficulties, I tend to quickly bounce back” (resilience), and “I am looking forward to the life ahead of me” (optimism). The items were rated on a 6-point scale (from 1 = strongly disagree to 6 = strongly agree). The PsyCap score was derived by averaging the scores across the four subscales. A higher score indicates greater psychological capital resources. The scale has demonstrated strong psychometric properties across diverse populations, including Czech, U.S., Slovak, and Japanese samples (Dudasova et al., 2021; Lorenz et al., 2022; Prochazka et al., 2023; Ikeda et al., 2023). Evidence supporting the applicability of the Chinese CPC-12R was reported by He (2024), who obtained good fit indices in confirmatory factor analysis (*CFI* = 0.942, *TLI* = 0.929, *RMSEA* = 0.049, *SRMR* = 0.057) and good internal consistency (α = *0.8*8). For the present sample, α = *0.9*0 was obtained. Moreover, the results of CFA suggest that the one-factor model has good fit indices (*CFI* = 0.928, *TLI* = 0.919, *RMSEA* = 0.050, *SRMR* = 0.061), confirming the construct validity of the scale.

#### 2.2.3 Creativity motivation

Creativity motivation was assessed via the Chinese adapted version of the Creativity Motivation Scale (CMS; He, 2023b), which was developed based on the understanding that creativity motivation is the driving force that compels individuals to participate in three domains of creative endeavors, including (1) learning, (2) experimentation, and (3) the pursuit of, innovative outcomes (Zhang et al., 2018). An example of the test items is “I feel pleasure when I bring a perceptible product to completion”. The participants responded on a 6-point Likert-type scale (1 = strongly disagree; 6 = strongly agree) to indicate the extent to which they agree to these statements. Previous studies have supported the reliability, validity, and applicability of the scale among Chinese students in Hong Kong (He, 2023b; Li et al., 2021). A high Cronbach's α = 0.87 was obtained in this study to support its internal reliability. Additionally, the fit indices from CFA support a one-factor model (CFI = 0.937, TLI = 0.904, RMSEA = 0.058, SRMR = 0.039) and confirm the construct validity of the scale.

#### 2.2.4 Creativity

##### 2.2.4.1 Idea production

Idea production was assessed via the Chinese WKCT (Wallach and Kogan, 1965; Cheung et al., 2004). Numerous studies have supported the reliability and validity of WKCT among Chinese students (Cheung and Lau, 2013; He, 2023a, 2024; He and Wong, 2021). In this study, the Chinese version of the WKCT comprises test items of both verbal and figural materials. The verbal component included alternate uses tasks, such as generating as many possible uses for a newspaper. The figural test items include pattern interpretation tasks, which require participants to generate as many meanings or associations as possible based on a given pattern. A total of 5 min were allowed to respond to each of the test items. Idea generation was scored by using two indices. First, fluency is scored by the total number of ideas. Second, flexibility is scored by the total number of categories into which the given ideas could be classified. Two experienced creativity researchers evaluated all the responses, and the average of their scores was included for data analysis. The intraclass correlation coefficients (ICCs) were all >0.90 (ICC_Verbal_fluency_ = 0.94; ICC_Verbal_flexibility_ = 0.93; ICC_Figural_fluency_ = 0.95; ICC_Figural_flexibility_ = 0.94; all *p*-values < 0.001), which suggested high interrater reliability. Moreover, the test showed good internal consistency in the current sample, with α = 0.83–0.86 (Table 1).

**Table 1:** Means, standard deviations, Cronbach's alpha coefficients, and correlation coefficients of the study variables.

**Variables**	***M***	***SD***	**α**	**1**	**2**	**3**	**4**	**5**	**6**	**7**	**8**	**9**	**10**	**11**	**12**	**13**
1. Age	21.5	1.91	–	1												
2. Gender	–	–	–	0.02	1											
3. Education	15.5	1.73	–	0.63^***^	0.04	1										
4. Dispositional mindfulness	3.88	1.42	0.86^**^	0.06	0.12^*^	0.06	1									
5. PsyCap	3.91	1.63	0.85^**^	0.07	0.07	0.07	0.58^***^	1								
6. Creative motivation	3.67	1.58	0.84^**^	0.04	0.09	0.08	0.08	0.59^***^	1							
7. WKCT-Fluency (verbal)	18.9	9.03	0.84^**^	0.08	0.13^*^	0.04	0.48^***^	0.44^***^	0.53^***^	1						
8. WKCT-Flexibility (verbal)	4.03	1.99	0.86^**^	0.07	0.12^*^	0.08	0.50^***^	0.46^***^	0.55^***^	0.37^***^	1					
9. WKCT-Fluency (figural)	20.1	10.4	0.83^**^	0.09	0.13^*^	0.05	0.52^***^	0.45^***^	0.55^***^	0.39^***^	0.48^***^	1				
10. WKCT-Flexibility (figural)	4.33	2.03	0.84^**^	0.06	0.12^*^	0.07	0.45^***^	0.48^***^	0.51^***^	0.40^***^	0.45^***^	0.44^***^	1			
11. TCT–DP	22.7	9.66	0.89^**^	0.04	0.12^*^	0.03	0.54^***^	0.49^***^	0.49^***^	0.17^**^	0.16^*^	0.18^*^	0.15^*^	1		
12. CPST (verbal)	66.9	9.78	0.81^**^	0.07	0.08	0.05	0.56^***^	0.51^***^	0.45^***^	0.14^*^	0.13^*^	0.15^*^	0.14^*^	0.13^*^	1	
13. CPST (figural)	64.3	10.7	0.80^**^	0.06	0.07	0.06	0.51^***^	0.47^***^	0.47^***^	0.15^*^	0.12^*^	0.14^*^	0.13^*^	0.15^*^	0.22^**^	1

Gender is dummy coded: 0 = male; 1 = female.

WKCT, Wallach-Kogan Creativity Test; TCT–DP, Test for Creative Thinking–Drawing Production; CPST, Creative Problem Solving Test.

^***^p < 0.001, ^**^p < 0.01, ^*^p < 0.05.

##### 2.2.4.2 Creative combination

Creative combination was measured via the Chinese translated version of the TCT–DP (He and Wong, 2011; Urban and Jellen, 1996), which was developed based on the holistic and componential approach to creativity. Previous studies have supported the reliability and validity of the test in Hong Kong Chinese students (He, 2023a, 2024). The test reflects creative combinatory ability via performance on a drawing task using an A4-sized sheet, which contains six distinct fragments in figural forms: (1) a small open square, (2) a broken line, (3) a curved line, (4) a 90° angle, (5) a semicircle, and (6) a point. Participants complete the drawing by assembling these elements in various ways, ranging from conventional, typical, separate, and simple designs to unconventional, atypical, complete, coherent, and visually sophisticated and compelling compositions. The test applies nine criteria for assessing creative performance: (1) continuation, (2) completion, (3) connections by line, (4) connections by theme, (5) new elements, (6) boundary breaking, (7) unconventionality, (8) perspective, and (9) humor and affectivity. The total score ranges from 0 to 66, with higher scores indicating stronger creative performance (see He and Wong, 2011, for detailed scoring procedures). In this study, the TCT–DP demonstrated excellent interrater reliability (ICC = 0.96) and high internal consistency (α = 0.90).

##### 2.2.4.3 Restructuring problem solving

The Chinese CPST (Lin et al., 2012; He and Wong, 2021) was used to evaluate creative problem solving via restructuring ability, specifically the “aha” (or sudden) insights of a novel and appropriate solution to a given problem (He, 2023b; Weisberg, 2015). The 10-item test consists of five verbal items and five figural items. The following is an example verbal test item: “How many cubic centimeters of dirt are in a hole 6 m long, 2 m wide, and 1 m deep?” Additionally, the following is an example figural test item: “Nine pigs are kept in a square pen. Build two more square enclosures that would put each pig in a pen by itself” (see He and Wong, 2021). A maximum of 20 min was allowed to complete the task. Problem-solving performance scores were determined by calculating the percentage of correctly answered verbal and figural problems. Previous research has confirmed the reliability, validity, and applicability of the test among Hong Kong and Taiwanese Chinese student samples (He, 2023b, 2024; He and Wong, 2021; Lin, 2023). In the current sample, the test demonstrated good internal consistency (α = 0.82 for verbal items; α = 0.83 for figural items).

### 2.3 Statistical analysis

Statistical analyses were performed with IBM SPSS version 28.0 for Windows and SPSS PROCESS macro 4.0 software (Hayes, 2013), with *p* < 0.05 indicating statistical significance. First, Pearson's correlations were calculated to examine the bivariate associations among the study variables as a preliminary step of the mediation analyses. Second, serial multiple mediation analyses were conducted via the SPSS PROCESS macro (Model 6; Hayes, 2013) to examine Hypotheses 1–3 regarding the sequential mediating effect of PsyCap and creativity motivation on the dispositional mindfulness–creativity link. Separate mediation models were built for each of the three aspects of creative performance. Ordinary least squares regression was used to calculate path coefficients for the total, direct, and indirect effects. A bias-corrected bootstrapping method with a resampling procedure of 5,000 samples was used to calculate the 95% confidence intervals (95% CIs) for the mediation effects. The indirect effect of the mediation path was considered statistically significant if the 95% CI did not include zero.

## 3 Results

### 3.1 Bivariate analyses

Table 1 presents the descriptive statistics and bivariate correlations of the key study variables. Significant correlations were observed between the predictor variable (dispositional mindfulness) and all assessed measures of the outcome variable (i.e., creative performance; *r*s = 0.45–0.56, all *p*-values < 0.01). Moreover, significant correlations were also found between the two hypothesized mediators and all the measures of creative performance [*r*s = 0.21–0.55 for PsyCap (*p*-values < 0.01), *r*s = 0.53–0.64 for creativity motivation (*p*-values < 0.001)]. With respect to the correlations between the predictor and the two mediators, dispositional mindfulness demonstrated a significant correlation only with the first mediator (PsyCap; *r* = 0.58, *p* < 0.001) but not with the second mediator (creativity motivation; *r* = 0.08, *p* = 0.76).

### 3.2 Mediation analyses

#### 3.2.1 Total effects and direct effects

**Figure 2:**
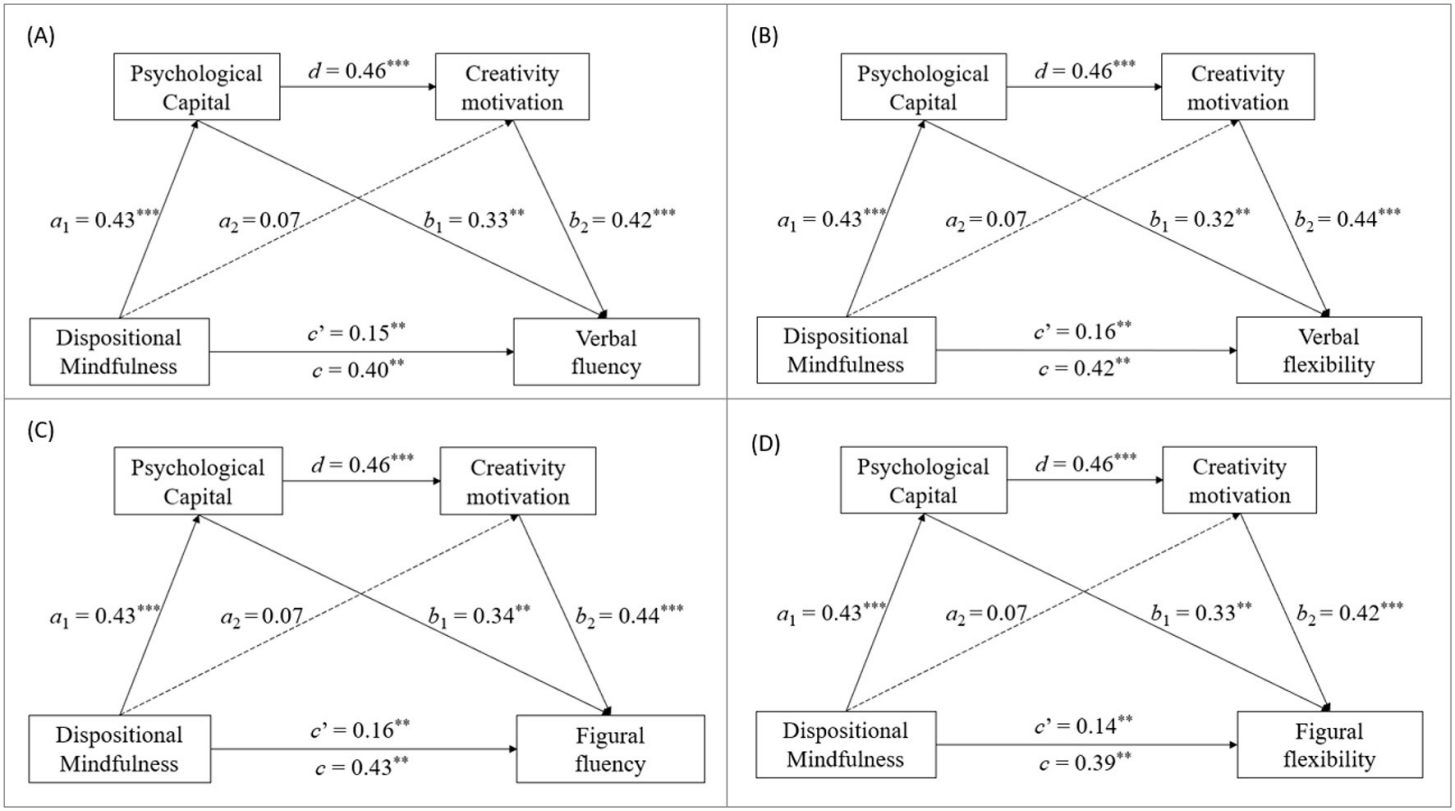
Results of the mediation analyses of the WKCT scores. ***p < 0.001; **p < 0.01.

**Figure 3:**
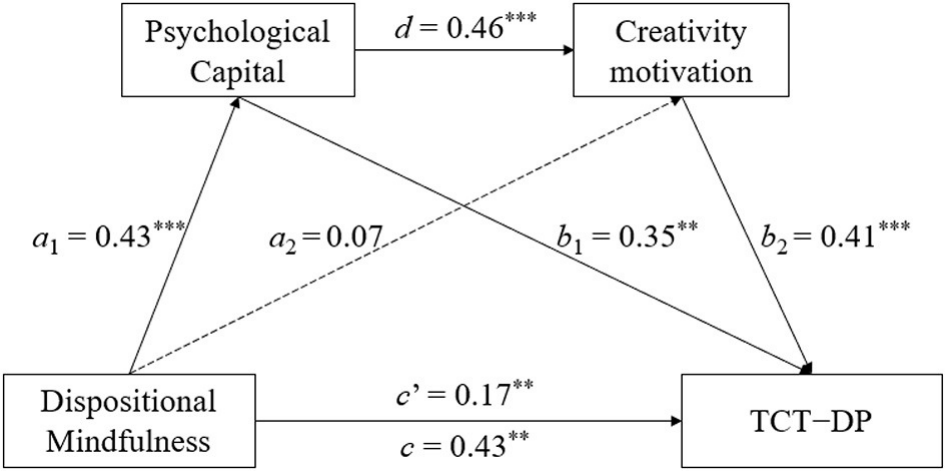
Results of the mediation analyses of the TCT-DP score. ***p < 0.001; **p < 0.01.

**Figure 4:**
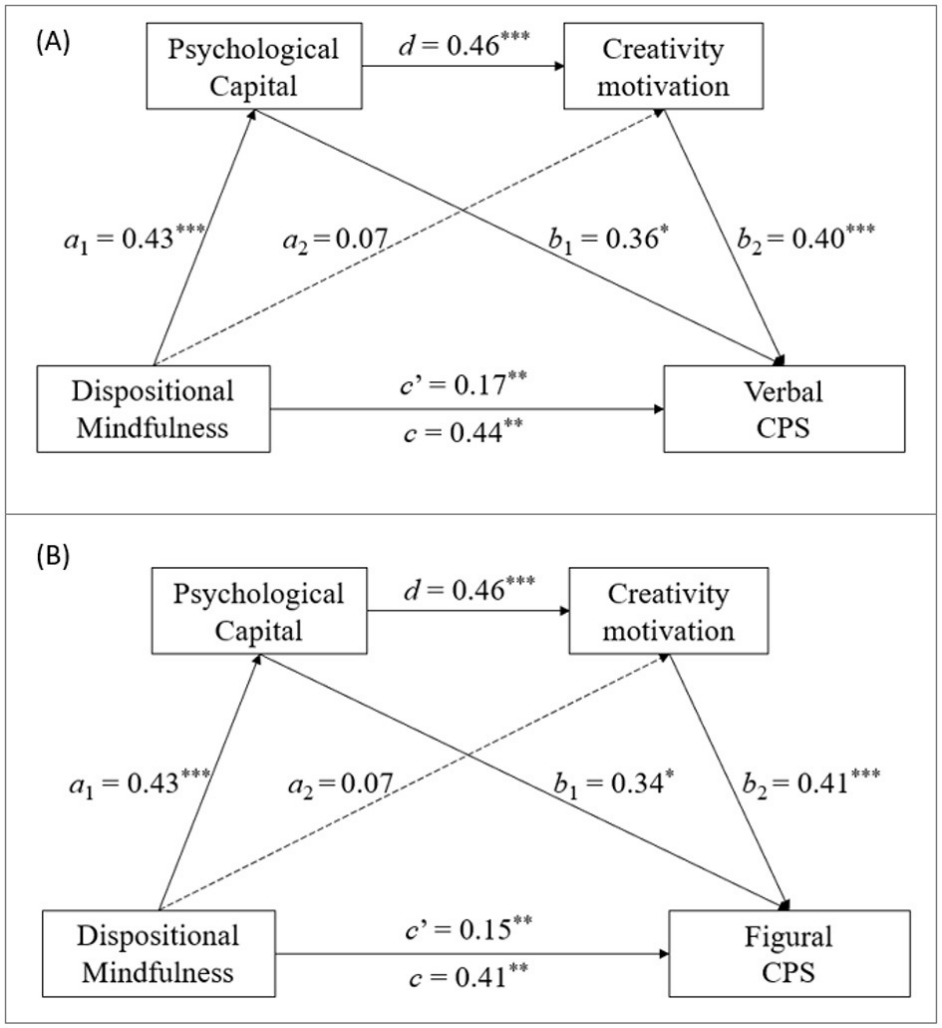
Results of the mediation analyses of Creative Problem-Solving (CPS) Test. ***p < 0.001, **p < 0.01, and *p < 0.05.

Figures 2–4 display the standardized coefficients of the multiple regression analyses regarding the direct paths among the study variables. The results indicated that dispositional mindfulness had a significant total effect (*c*) on all three dimensions of creative performance. This included the four indices of idea production assessed by the WKCT (*c* = 0.39–0.43, *p* < 0.01; Figure 2), creative combination measured by the TCT-DP (*c* = 0.43, *p* < 0.01; Figure 3), and restructuring problem-solving evaluated through the CPST (*c* = 0.41–0.44, *p* < 0.01; Figure 4).

Moreover, the direct effects of dispositional mindfulness on the three dimensions of creative performance were statistically significant (*c*′ = 0.14–0.16, *c*′ = 0.17, and *c*′ = 0.15–0.17 for idea production, creative combination, and restructuring problem solving, respectively; all *p*-values < 0.05). Statistical significance was also found for the path between the two mediators of the connection from PsyCap to creativity motivation (*d* = 0.46, *p* < 0.001). However, the direct effects of dispositional mindfulness on the two mediators were significant only for the path from dispositional mindfulness to PsyCap (mediator 1; *a*_1_ = 0.43, *p* < 0.01) but not for the path from dispositional mindfulness to creativity motivation (mediator 2; *a*_2_ = 0.07, *p* = 0.35) in all the models. Among the paths from the two mediators to creative performance, all the paths from PsyCap (mediator 1; *b*_1_ = 0.32–0.34, *p*-values < 0.001) and from creativity motivation (mediator 2; *b*_2_ = 0.42–0.44, *p*-values < 0.001) to all four indices of idea production were statistically significant. Moreover, the paths from PsyCap (*b*_1_ = 0.35, *p* < 0.01) and creativity motivation (*b*_2_ = 0.41, *p* < 0.001) to creative combination were statistically significant. Additionally, for restructuring problem solving, the paths from PsyCap (*b*_1_ = 0.34–0.36, *p*-values < 0.05) and creativity motivation (*b*_2_ = 0.40–0.41, *p*-values < 0.001) to both verbal and figural restructuring problem solving were significant.

#### 3.2.2 Indirect effects

The results of the indirect effects are summarized in Tables 2–4.

**Table 2:** The indirect effects of perceived school climate on the WKCT scores via creative self-efficacy and creativity motivation.

**Effect**	**Pathway**	**Bootstrap estimate**		**95% CI**		**P_M_**
		β	**SE**	**LL**	**UL**	
Total IE	IE1+ IE2 + IE3	0.25	0.14	0.11	0.43	62.5%
IE1	Mindfulness → PsyCap → verbal fluency (*a*_1_ × *b*_1_)	0.14	0.10	0.03	0.27	35.0%
IE2	Mindfulness → creativity motivation → verbal fluency (*a*_2_ × *b*_2_)	0.03	0.08	0.01	0.10	7.50%
IE3	PSC → PsyCap and creativity motivation → Verbal fluency (*a*_2_ × *d* × *b*_2_)	0.08	0.06	0.02	0.19	20.0%
Total IE	IE1+ IE2 + IE3	0.26	0.18	0.11	0.40	61.8%
IE1	Mindfulness → PsyCap → verbal flexibility (*a*_1_ × *b*_1_)	0.14	0.09	0.04	0.26	33.3%
IE2	Mindfulness → creativity motivation → verbal flexibility (*a*_2_ × *b*_2_)	0.03	0.07	0.00	0.17	7.14%
IE3	Mindfulness → PsyCap and creativity motivation → verbal flexibility (*a*_2_ × *d* × *b*_2_)	0.09	0.08	0.01	0.20	21.4%
Total IE	IE1+ IE2 + IE3	0.27	0.16	0.13	0.49	62.8%
IE1	Mindfulness → PsyCap → figural fluency (*a*_1_ × *b*_1_)	0.15	0.12	0.06	0.27	34.9%
IE2	Mindfulness → creativity motivation → figural fluency (*a*_2_ × *b*_2_)	0.03	0.04	0.01	0.19	6.98%
IE3	Mindfulness → PsyCap and creativity motivation → figural fluency (*a*_2_ × *d* × *b*_2_)	0.09	0.07	0.03	0.23	20.9%
Total IE	IE1+ IE2 + IE3	0.25	0.14	0.12	0.54	64.1%
IE1	Mindfulness → PsyCap → figural flexibility (*a*_1_ × *b*_1_)	0.14	0.12	0.02	0.29	35.9%
IE2	Mindfulness → creativity motivation → figural flexibility (*a*_2_ × *b*_2_)	0.03	0.06	0.00	0.18	7.69%
IE3	Mindfulness → PsyCap and creativity motivation → figural flexibility (*a*_2_ × *d* × *b*_2_)	0.08	0.09	0.03	0.26	20.5%

WKCT, Wallach-Kogan Creativity Test; IE, indirect effect; PSC, perceived school climate; P_M_, proportion of the total effect accounted for.

In support of Hypothesis 1, the results in Table 2 suggest that the indirect effects of dispositional mindfulness on the four indices of idea production through the first mediator (i.e., creative self-efficacy; *a*_1 ×_ *b*_1_) were statistically significant (β = 0.14–0.15, *SE* = 0.08–0.10; all 95% CIs did not include 0). Moreover, the indirect effects through the second mediator (i.e., creativity motivation; *a*_2 ×_ *b*_2_) were also statistically significant (all β values = 0.03, *SE* = 0.01–0.02, all 95% CIs did not include 0). Furthermore, the indirect effects through the serial mediating effect of PsyCap and creativity motivation (i.e., *a*_2 ×_ *d* × *b*_2_; β = 0.08–0.09, *SE* = 0.04–0.06, all 95% CIs did not include 0) were statistically significant. The percentages of the total indirect effects mediated by the two mediators were 62.5%, 61.8%, 62.8%, and 64.1% for verbal fluency, verbal flexibility, figural fluency, and figural flexibility, respectively.

In support of Hypothesis 2, the results in Table 3 reveal that the indirect effects of dispositional mindfulness on creative combination performance, as measured by the TCT–DP score, were mediated through the first mediator, PsyCap [β = 0.15, *SE* = 0.06, 95% CI (0.02, 0.23)], and through the second mediator, creativity motivation [β = 0.03; *SE* = 0.01, 95% CI (0.00, 0.18)], were statistically significant. Moreover, the indirect effect through the serial mediating effect of the two mediators [β = 0.08; *SE* = 0.04, 95% CI (0.01, 0.22)] was also significant. The percentage of the total indirect effect mediated by the combination of the two mediators was 60.5%.

**Table 3:** The indirect effects of perceived school climate on the TCT–DP score via creative self-efficacy and creativity motivation.

**Effect**	**Pathway**	**Bootstrap estimate**		**95% CI**		**P_M_**
		β	**SE**	**LL**	**UL**	
Total IE	IE1+ IE2 + IE3	0.26	0.13	0.12	0.48	60.5%
IE1	Mindfulness → PsyCap → TCT–DP (*a*_1_ × *b*_1_)	0.15	0.11	0.02	0.23	34.9%
IE2	Mindfulness → creativity motivation → TCT–DP (*a*_2_ × *b*_2_)	0.03	0.05	0.00	0.18	6.98%
IE3	Mindfulness → PsyCap and creativity motivation → TCT–DP (*a*_2_ × *d* × *b*_2_)	0.08	0.07	0.01	0.22	18.6%

TCT–DP, Test for Creative Thinking–Drawing Production; IE, indirect effect; PSC, perceived school climate; P_M_, proportion of the total effect accounted for.

In support of Hypothesis 3, the results in Table 4 suggest that the indirect effects from dispositional mindfulness to both the verbal [β = 0.16, *SE* = 0.04, 95% CI (0.01, 0.20)] and figural [β = 0.15, *SE* = 0.06, 95% CI (0.04, 0.29)] CPST scores through the first mediator (i.e., PsyCap) were statistically significant. Moreover, the indirect effects through the second mediator (i.e., creativity motivation) were also significant for both the verbal [β = 0.03, *SE* = 0.01, 95% CI (0.01, 0.15)] and figural [β = 0.03, *SE* = 0.01, 95% CI (0.00, 0.21)] scores. Furthermore, the indirect effects through the serial mediating effect of the two mediators (β = 0.08, *SE* = 0.02, all 95% CIs did not include 0) were also statistically significant. The percentages of the total indirect effect mediated by the combination of the two mediators were 61.4 and 63.4% for verbal and figural restructuring problem solving, respectively.

**Table 4:** The indirect effects of perceived school climate on the CPST scores via creative self-efficacy and creativity motivation.

**Effect**	**Pathway**	**Bootstrap estimate**		**95% CI**		**P_M_**
		β	**SE**	**LL**	**UL**	
Total IE	IE1+ IE2 + IE3	0.27	0.13	0.07	0.39	61.4%
IE1	Mindfulness → PsyCap → verbal CPS (*a*_1_ × *b*_1_)	0.16	0.09	0.01	0.20	36.4%
IE2	Mindfulness → creativity motivation → verbal CPS (*a*_2_ × *b*_2_)	0.03	0.06	0.01	0.15	6.82%
IE3	Mindfulness → PsyCap and creativity motivation → verbal CPS (*a*_2_ × *d* × *b*_2_)	0.08	0.10	0.01	0.18	18.2%
Total IE	IE1+ IE2 + IE3	0.26	0.11	0.03	0.41	63.4%
IE1	Mindfulness → PsyCap → figural CPS (*a*_1_ × *b*_1_)	0.15	0.10	0.04	0.29	36.6%
IE2	Mindfulness → creativity motivation → figural CPS (*a*_2_ × *b*_2_)	0.03	0.08	0.00	0.21	7.32%
IE3	Mindfulness → PsyCap and creativity motivation → figural CPS (*a*_2_ × *d* × *b*_2_)	0.08	0.10	0.02	0.25	19.5%

CPST, creative problem-solving Test; IE, indirect effect; CPS, creative problem solving; P_M_, proportion of the total effect accounted for.

## 4 Discussion

### 4.1 Theoretical significance

This study advances the theoretical understanding of the mindfulness–creativity relationship by identifying the sequential mediating roles of PsyCap and creativity motivation in this association. While prior research has demonstrated a direct link between mindfulness and creativity (Henriksen et al., 2020; He, 2023a), research into the mechanisms underlying this relationship has only recently emerged (He, 2024), and further empirical scrutiny is needed to increase the understanding of these mechanisms. By emphasizing the pivotal role of PsyCap as a primary psychological resource and task-related motivation as a subsequent psychological resource that sustains the effect of PsyCap, this study aligns with and extends the principles of positive psychology, which highlights the importance of personal strengths and resources in facilitating optimal human functioning (Seligman and Csikszentmihalyi, 2000). These findings support the framework that bridges mindfulness, psychological resources, and motivational pathways, offering a comprehensive perspective on how these elements interact to influence creative functioning. The finding that PsyCap plays a strong mediating role in the relationship between mindfulness and creativity motivation is particularly significant. This effect underscores the centrality of psychological resources, such as hope, self-efficacy, resilience, and optimism, in translating positive traits such as dispositional mindfulness into motivational outcomes. This finding supports theoretical models such as COR theory, which posits that personal resources enable individuals to sustain motivation and engagement during challenging tasks (Hobfoll et al., 2018). Moreover, this result complements previous studies suggesting that PsyCap enhances creativity through its positive impact on cognitive and emotional regulation (Sun et al., 2023; He, 2024). The multidimensional nature of PsyCap, which integrates emotional resilience and task-focused optimism, provides a nuanced understanding of how psychological strengths act as bridges among positive traits, cognitive and emotional regulation, and behavioral outcomes.

Furthermore, as an interesting finding that warrants a discussion, the study revealed no direct relationship between dispositional mindfulness and creativity motivation. This finding refines prior research that emphasized the broader psychological benefits of trait mindfulness, such as its positive associations with self-talk and emotional regulation (Grzybowski and Brinthaupt, 2022). This finding also suggests that mindfulness may require intermediary factors, such as PsyCap, to be translated into domain-specific motivational outcomes. This perspective highlights the importance of context and mediators when examining the motivational effects of mindfulness. By challenging existing assumptions about the direct influence of mindfulness on creativity motivation, this study contributes to a more precise delineation of its boundaries and effects. Moreover, the multidimensional approach to assessing creativity—examining idea production, creative combination, and restructuring problem solving—adds depth and breadth to the findings. The mediating roles of PsyCap and creativity motivation were consistent across all three dimensions, reinforcing the robustness and generalizability of the proposed model. Consistent with the studies by He (2023a, 2024), these findings support the application of a multidimensional framework in the conceptualization of creativity, which enriches the understanding of the mindfulness–creativity relationship by accounting for the complex and multifaceted nature of creativity (Sternberg et al., 2024; Runco, 2024). Furthermore, the findings of this study highlight the need for future research to explore additional mediators or contextual moderators, such as cultural influences or task-specific demands, that may further clarify the intricate dynamics of mindfulness and creativity.

As another interesting finding that requires attention, although the sequential mediation model was supported, the presence of a significant direct effect from dispositional mindfulness to creativity, even after accounting for PsyCap and creativity motivation, suggests that the model is insufficient to fully capture all relevant psychological mechanisms underlying the mindfulness-creativity relationship. In fact, the partial mediation was theoretically expected, given a certain level of the conceptual overlap among constructs such as mindfulness, psychological resources, and creativity (He, 2024; Hughes et al., 2023). However, the finding regarding the residual direct path is particularly interesting, which implies the possibility that dispositional mindfulness may also influence creativity through other unexamined mechanisms. For example, emotion regulation, which is frequently linked to mindfulness, may independently enhance creative thinking through the pathways of promoting cognitive flexibility and facilitating idea generation (Grzybowski and Brinthaupt, 2022; Sun et al., 2023). In addition, personality traits such as openness to experience may interact with trait mindfulness to predict creative behavior, particularly in novel or uncertain environments (Tsai et al., 2024). Furthermore, contextual factors such as perceived autonomy support from living or working environments may moderate how trait mindfulness translates into creativity-related outcomes (Wu and Lee, 2020). These considerations indicate that the relationship between mindfulness and creativity is likely multifaceted and multidimensional; rather than strictly sequential. Future studies may enrich this line of research by incorporating additional mediators (e.g., emotional intelligence, cognitive resources) and potential moderators (e.g., autonomy support, cultural values, domain-specific demands) to generate a more complete understanding of the mechanisms underlying the mindfulness–creativity link.

In summary, this study enriches the theoretical understanding of the mindfulness–creativity association by integrating PsyCap and creativity motivation into a cohesive framework and using a multi-assessment approach to measure creative outcomes. It highlights the interplay among cognitive, affective, mental, and motivational dimensions in explaining how dispositional mindfulness facilitates creative functioning. Importantly, the finding of partial mediation underscores the need to explore additional mechanisms and boundary conditions that may shape this relationship. These insights open avenues for future research that aims to refine theoretical models and identify the broader contextual, dispositional, emotional, mental, and self-regulatory factors that may contribute to creativity.

### 4.2 Practical significance

The findings of this study provide valuable insights into enhancing creativity among university students by integrating mindfulness practices with the development of PsyCap and creativity motivation. These practical implications emphasize the importance of fostering both psychological resources and motivational drivers to unlock students' creative potential in educational settings. The mediating role of PsyCap highlights its importance as a foundational resource for creativity. For university students, who often face academic challenges and problem-solving demands, fostering PsyCap can increase the resilience, optimism, and self-efficacy needed to sustain creative engagement. PsyCap can be effectively developed through structured group activities and collaborative problem solving, which aligns with its theoretical framework (Luthans et al., 2007, 2024; He, 2024). These activities provide opportunities for students to engage in goal-oriented tasks, overcome challenges collaboratively, and reflect on successes, thereby reinforcing the core components of PsyCap, such as hope, self-efficacy, resilience, and optimism. Mindfulness practices, such as meditation or reflective journaling, can also be incorporated into academic settings to indirectly support creativity by strengthening psychological resources. The study highlights that trait mindfulness alone does not directly increase creativity motivation; rather, it works through PsyCap as an intermediary. These findings suggest that universities should combine mindfulness training with PsyCap-focused initiatives. For example, mindfulness exercises aimed at increasing present-moment awareness can be paired with activities fostering hope and resilience, such as envisioning future successes or reflecting on personal growth. These integrative practices align with findings that mindfulness facilitates emotional regulation and increases psychological resources, which in turn support creative engagement (Tran et al., 2021).

Tailored interventions can address specific dimensions of creativity, such as idea production, creative combination, and restructuring problem solving. Brainstorming sessions and fluency exercises can encourage students to generate multiple ideas and divergent production, whereas concept mapping or interdisciplinary projects can help them synthesize unrelated ideas into innovative solutions. Real-world case studies and simulation exercises can challenge students to apply their restructuring ability and creativity to practical problems (Sternberg et al., 2024; Runco, 2024). These targeted approaches ensure that creativity-related interventions are both effective and aligned with the diverse needs of students in higher education. Furthermore, universities can integrate creativity development into existing curricula by embedding PsyCap and mindfulness training into coursework and extracurricular activities. For example, general education courses could include modules on creative thinking and problem solving combined with mindfulness exercises to increase focus and emotional regulation. Extracurricular programs, such as innovation labs or student-led projects, can provide opportunities for students to apply PsyCap and creativity motivation in collaborative settings. These programs can benefit from systematic assessments of students' creative outcomes (He, 2023a) via tools and methods designed to align with educational goals. Additionally, fostering a supportive educational environment that nurtures students' psychological and motivational resources is crucial. Educators and administrators can achieve an environment by providing constructive feedback, recognizing creative achievements, and encouraging intellectual risk-taking. For example, peer mentoring programs in which experienced students guide their peers through creative challenges have been shown to enhance both PsyCap and creativity motivation (Tran et al., 2021). A culture of collaboration and inclusion encourages students to explore and express their creativity without fear of judgement.

In summary, the findings of this study offer a practical framework for increasing creativity in university students by combining mindfulness practices with strategies to develop PsyCap and creativity motivation. These interventions, when integrated into educational settings, can empower students to achieve their creative potential and prepare them for future academic and professional success.

### 4.3 Limitations and directions for future research

Despite its contributions, this study has several limitations that warrant consideration. First, the cross-sectional design of the study precludes causal inferences. While the mediation model provides evidence for the hypothesized relationships, longitudinal or experimental studies are needed to confirm the directionality of the effects. Second, the study sample, which was composed of university students in Hong Kong, may limit the generalizability of the findings to other populations and cultural contexts. Cultural factors, such as the emphasis on collectivism and conformity in East Asian societies, may influence the relationships among mindfulness, PsyCap, and creativity. Cross-cultural research could investigate whether similar mechanisms operate in individualistic cultures. Moreover, the current sample comprises only undergrade students in the disciplines of education, sciences, health sciences, social sciences, linguistics, or arts. Future study may generalize the research findings to other student populations (e.g., postgraduate students, primary and secondary school students) and students from other disciplines, such as business or engineering. Third, the study did not examine potential moderators, such as personality traits or environmental factors, that could influence the strength of the relationships in the model. For example, openness to experience or workplace support might improve the link between mindfulness and PsyCap or between PsyCap and motivation. Finally, while the multiple-measurement approach provides a comprehensive assessment of creativity, it may not fully capture domain-specific or real-world creative performance. Future research could incorporate task-based assessments or field studies to validate these findings in practical settings.

## 5 Conclusion

This study advances the understanding of how trait mindfulness fosters creativity by revealing the sequential mediating role of PsyCap and creativity motivation. The findings highlight PsyCap as a critical resource that bridges the gap between mindfulness and creativity, emphasizing the importance of positivity-oriented resource-building interventions. Moreover, the absence of a direct link between mindfulness and creativity motivation underscores the complexity of the motivational processes in the mindfulness–creativity link. By addressing these nuances, this research provides a foundation for future exploration and practical applications with respect to the roles of positive personal attributes, psychological resources, and creative functioning in educational and organizational contexts.

## Data Availability

The datasets presented in this article are not readily available because the raw data analyzed in this study are not publicly available due to ethical restrictions and confidentiality agreements but are available from the corresponding author upon reasonable request. Requests to access the datasets should be directed to mavishe@eduhk.hk.
